# Foundations of Intervention Research in Instrumental Practice

**DOI:** 10.3389/fpsyg.2015.02014

**Published:** 2016-01-22

**Authors:** Johannes L. Hatfield, Pierre-Nicolas Lemyre

**Affiliations:** ^1^Department of Music Education, Norwegian Academy of MusicOslo, Norway; ^2^Department of Coaching and Psychology, Norwegian School of Sport SciencesOslo, Norway

**Keywords:** intervention, mental practice, performance profile, practice journal, self-determination

## Abstract

The goals of the present study are to evaluate, implement, and adapt psychological skills used in the realm of sports into music performance. This research project also aims to build foundations on how to implement future interventions to guide music students on how to optimize practice toward performance. A 2-month psychological skills intervention was provided to two students from the national music academy's bachelor program in music performance to better understand how to adapt and construct psychological skills training programs for performing music students. The program evaluated multiple intervention tools including the use of questionnaires, performance profiling, iPads, electronic practice logs, recording the perceived value of individual and combined work, as well as the effectiveness of different communication forms. Perceived effects of the intervention were collected through semi-structured interviews, observations, and logs.

## Introduction

In music, the quality of practice and the level of performance are intricately linked to one another. The famous pianist and pedagogue, Walter Gieseking, pointed out a central aspect of teaching music: “*one of the most important duties of a pedagogue, if not the most important, is to teach the pupil how to practice correctly*” (Leimer and Gieseking, 1972, p. 46). However, during the last four decades, several studies revealed that music students are recurrently not provided with information about how to practice, but how to play and perform the music (Jørgensen, 1996; Jørgensen and Lehmann, 1997; Atkins, 2009; Gaunt, 2009; Lehmann and Jørgensen, 2012; Burwell and Shipton, 2013). Consequently, guiding the learning of the art of instrument practice appears to be both underestimated and neglected in higher music education. Important issues such as how to plan and organize instrument practice, what sort of goals enhance progress and motivation to practice, how to solve specific tasks, or even how to evaluate instrument practice are rarely addressed within the context of higher music education. Findings from different research studies imply that performing music students tend to lack proactivity toward the planning of physical and mental practice (Jørgensen, 1996; Gaunt, 2009; Lehmann and Jørgensen, 2012; Burwell and Shipton, 2013). Several studies have indicated a potential for individual development during higher music education (Ericsson et al., 1993; Jørgensen, 1996, 2000; Nielsen, 2004; Ericsson, 2006). These studies have all noted that there is a need to help music students to utilize their time and effort in ways that motivate and enable stable progress toward successful professional music careers. In a study investigating health and wellbeing related issues in seven British conservatories revealed that most students expressed a general need for one-to-one teaching in relation to performance and practice issues, injury prevention, and the development of professional skills (Atkins, 2009). These findings all support an imperative need in music students to be formally guided on how practice.

Contrastingly, the field of sports has for many years been concerned about developing and sustaining the best possible training environments to facilitate optimal performance in athletes (Starkes and Ericsson, 2003). Setting goals, applying visualization skills in training and competition, developing self-regulation skills and monitoring training with the help of training logs, implementing injury and dropout prevention protocols in combination with other mental techniques have become an important part of the developmental routine used by aspiring and established athletes (Kyllo and Landers, 1995; Lemyre, 2005; Dosil, 2006; Gjerset, 2008; Hanrahan and Andersen, 2010; Weinberg and Gould, 2011). Among other things, mental training for sports typically emphasizes psychological factors such as personal mastery of tasks, intrinsic motivation, and competing with oneself through goal-directed activities (Andersen, 2005; Dosil, 2006; Hanrahan and Andersen, 2010; Weinberg and Gould, 2011).

Within the music performance context, the phenomenon of mental practice is related to mental rehearsal, which indicates the action of visualizing the music score and creating various sensory images of how to perform a piece of music without muscular movements (Coffman, 1990). However, in the present study, mental practice is conceptualized in accordance with the definition of psychological skills training (PST) within the field of sports psychology; that is, a “*systematic and consistent practice of mental or psychological skills for the purpose of enhancing performance, increasing enjoyment, or achieving greater sport and physical self-satisfaction”* (Weinberg and Gould, 2011, p. 248).

PST-techniques have been considered through almost five decades of research within sport psychology. Techniques such as goal setting, arousal regulation, visualization, concentration, and internal dialog in relation to peak performance have been highlighted (Locke, 1968; Locke et al., 1981; Orlick and Partington, 1988; Beauchamp et al., 1996; Andersen, 2000; Papaioannou et al., 2004; Pavlidou and Doganis, 2008; Burton et al., 2010). When introducing PST to the context of music performance and practice, assessing intervention tools and adapting them to the music context is vital to ensure their relevance and effectiveness (Hays, 2002). This study explicitly focuses on the process of tailoring a PST-program evaluating intervention tools in collaboration with two performing music students. The intervention tools evaluated were: Use of questionnaires, performance profiling, iPads/practice application, combined individual and group PST-sessions and communication emphasizing self-reference and choice.

The study was based on the following hypotheses:

1. Self-assessment through questionnaires and performance profiling motivates students for further work through identification of individual key-issues.
2. Music students utilizing iPads with a practice application keeping track of accumulated practice with a diary and video-recording possibility, experience improvement in concentration and self-reflection toward music practice.
3. PST-intervention is best implemented through a combination of individual and single-group sessions.
4. Implementation of PST feels intrinsically motivating, emphasizing self-referenced learning through non-controlling communication as each participant's personal development and experience of the program is highlighted throughout the intervention.

## Background

Few studies have investigated music performance and practice acquisition from an intervention standpoint. Clark and Williamon (2011) found that a 9-week musician-specific mental training program increased students' self-efficacy, practice behavior, imagery vividness, self-awareness, and self-confidence, and incited healthier perspectives toward making music. Issues concerning the content and delivery of the program revealed that participants felt that the program could have been strengthened through more examples of practical application, case studies, and a greater use of class discussion to increase experience sharing and knowledge transfer (Clark and Williamon, 2011). Several other studies introducing mental training to music students revealed similar findings. In a study by Hoffman and Hanrahan (2012), music students were provided three 1-h psycho-educational workshops. In a 1-month follow up assessment, a significant increase in students' performance quality and a significant decrease in performance anxiety in the intervention group were revealed. Another study by Osborne et al. (2014) introducing performance psychology techniques to music students over a 3-week period revealed similar results. After a 3-weeks intervention, students reported a significant reduction of self-reported music performance anxiety. The intervention also enhanced student's performance, preparation, confidence, courage, focus, concentration, and performance resilience (Osborne et al., 2014). Other types of interventions such as yoga (Khalsa and Cope, 2006) cognitive behavioral therapy (Kendrick et al., 1982) and Alexander technique (Valentine et al., 1995) have also revealed positive effects on reducing performance anxiety in music students and performers. These studies targeted music performance enhancement and music performance anxiety reduction. Burwell and Shipton (2013) recently conducted an action research project regarding strategic approaches to practice, which applied an individual intervention approach. Eight performing music students undertook a 2-week practice clinic for practice seminars, group discussions, practicing, and self-reflection. Their participation in the practice clinic generated interesting insights regarding individual practice, planning, structure, time-management, and metacognitive thinking. Moreover, findings revealed that music students, to their own surprise, had comparatively little knowledge about instrument practice and practice strategies before entering the clinic. The authors found that the students had little insight as to what motivated them to pursue their music training and what factors influenced the quality outcome of their practice.

Motivation research has clearly established the importance of autonomy support and self-reference in learning (Zimmerman et al., 1992; Deci and Ryan, 2000; Locke and Latham, 2002; Elliot and Dweck, 2005; Zimmerman, 2008). Moreover, when behavior or communication forms used by teachers, coaches or instructors is intended to exert control over their pupil it is likely to lead to a significant loss and lack of interest in the associates task (Deci and Ryan, 2000). Using words such as must, ought to, have to, or explaining the assumed right way deductively may be perceived as controlling. However, when the learning context is characterized by an emphasis on explicit rationales, the learner's voice, and a high degree of self-determination, it is likely to foster a high quality in motivation (Deci and Ryan, 1985; Locke and Latham, 1990). Accordingly, both inductive and deductive forms of communication might positively affect the quality of motivation as long as individuals perceive the learning context as congruent with one's intrinsic values (Deci and Ryan, 2000). The present intervention deliberately applied autonomy-supportive and non-controlling forms of communication emphasizing explicit purpose for implementing PST, using open-ended questions, supporting autonomy and encouraging self-reference.

## Theory

The present study was conceptually based on two theories, Self-regulation theory (SRT), Zimmerman (2002), and Deci and Ryan's (1985, 2000) Self-determination theory (SDT).

Experienced-based literature on music practice yields similarities with psychological skills used in sports and within the literature on deliberate practice (Ericsson et al., 1993; Ericsson, 2006; Weinberg and Gould, 2011). The famous Russian pianist and pedagogue Genrikh Gustavovich Neuhaus introduced his work on music practice in the following fashion: “*Mastery of the art of working, of learning composition- which is one of the reliable criteria of a pianists ‘maturity- is characterized by an unwavering determination and an ability not to waste time. The greater the part played in this process but willpower (going straight to the goal) and concentration, the better result. The greater the passivity and inertia- the greater the time for learning a composition, while interest in it inevitably flag. All this is well known, but to repeat it is not useless*” (Neuhaus, 1993, p. 4). Self-regulation is viewed in relation to what we know as deliberate practice, which is basically the study of how experts become experts. Self-regulation theory emphasizes deliberate work strategies such as planning and goal setting, meta-analytic practice, evaluation, and self-reflection (Zimmerman and Schunk, 1989; Zimmerman and Risemberg, 1997; Zimmerman, 2002). Furthermore, the context in which music students find themselves might have a huge impact on the actual quality of motivation, and thus, how instrument practice and performance is executed. Consequently, establishing an environment that nourishes autonomous motivation is an essential goal of the current evaluation study. The present study is conceptually based on Self-Determination Theory (SDT; Deci and Ryan, 1985, 2000). SDT is based on three *basic psychological needs*, relatedness, autonomy, and competence. When these three needs are fulfilled, *need-satisfaction* takes place. Moreover, SDT claims that the degree to which these needs are satisfied will impact one's well-being, life satisfaction, volition, and quality of motivation (Ryan and Deci, 2000; Deci and Ryan, 2008). Furthermore, when the learning context is dominated by external regulations such as demands, deadlines, incentives, and other contingencies, students are more likely to be less intrinsically motivated and self-driven. When the learning context is characterized by satisfaction of the three basic psychological needs, motivation is most likely to reach the most integrated level, *identified regulation*. Thus, the more a task is controlled by external factors, the less identification, integration, and intrinsic motivation is experienced on the task (Deci et al., 1999; Deci and Ryan, 2000). Examples of contrasting types of external regulations are illustrated in the following scenarios:

1. Scenario 1: If a music student is forced to play a piece of music, which he dislikes, the student is most likely to feel unmotivated and bored while practicing it. This is by SDT referred to as *controlled regulation* as the student may still perform the task to pass a course.
2. Scenario 2: The same student is introduced to the same piece of music but additionally provided with a rationale for why this piece might be especially beneficial to personal artistic development and at the same time given the autonomy to choose another piece to practice. The student would likely start to identify with the new pieces, *identified regulation*, and at the same time feel autonomous, respected and supported by the context in which learning takes place. Moreover, this indicates that music students who have a basic intrinsic motivation to play a musical instrument might be receptive to external regulation of instrument practice if the regulation resonates with their sense of self. Consequently, when students identify the usefulness of an exercise such as goal setting, the exercise becomes internalized through “*transforming the regulation into their own so that it will emanate from their sense of self*” (Ryan and Deci, 2000, p. 60).

The present study will seek to generate explorative empirical data on how to tailor a functional PST program for performing music students through the conceptual lens of SDT (Deci and Ryan, 2000). It will aim to offer insight as to *what are the key elements to include in a functional psychological skills program (PST) for musicians to promote lasting motivation, progress, and deliberate practice?*

## Material and methods

A 2-month PST intervention was provided to two students attending the last year of their Bachelor degree in music performance during the second half of the fall semester.

The main components evaluated in this study were the use of questionnaire in combination with performance profiling, use of iPads and electronic practice journal, the use of individual and group settings, and use of non-controlling communication enabling participants' self-determined activities.

### Participants and procedures

Two fourth-year bachelor students (using pseudonyms), Marcus 22, violinist, and Rita 21, pianist, were voluntarily recruited from the music performance program at the music academy. A letter of recruitment was sent electronically to 34 bachelor level performance students at the academy. Due to broad interest, recruitment was finally based on a combination of participants' interest, availability and time. Their teachers described the participants as average achievers when compared to fellow students.

The intervention was organized into weekly 60-min sessions, two group meetings and two individual meetings per month. Altogether, the two participants attended four individual sessions and four group sessions. The rationale for varying the sessions was to stimulate both individual progress and group reflection throughout the program. In addition, the group sessions were intended to serve as an arena in which the students could display their progress every other week, while gaining feedback and reflecting on further progress and goals.

The first week of intervention comprised of individual assessment based on questionnaires sent by mail 1 week prior to the first individual meeting. After completion, questionnaire scores where transcribed into performance profiles providing the participants with an individual and visual impression of current performance and practice level. During the first meeting, an individualized semi-structured interview was conducted recording participants' opinions of current level of performance and personal impression of the completed questionnaires. Perceived focus points, individual short and long-term goals were identified. Goal setting informed the choice of strategies and tools implemented by the students throughout the program. Meetings generated information on the perceived usefulness of intervention tools as well as providing participants with PST. Consequently, the participant's awareness increased in relationship to their own practice and the intervention process. Main interactions from each session were recorded and transcribed shortly after every session. The research log documented the participants' views, discussions, and experiences from 1 week to the next throughout the intervention. Meetings consisted of questions about the participants' progress, perceived goal achievement, and accumulated practice specific to intervention tools. The other half of the sessions consisted of discussions pertaining to concrete strategies, goal setting, and trying out new mental skills that could be added to participants' practice routines and performances. Researchers' reflections based on observations concerning use of intervention tools were also integrated to the research log.

Two main focus areas were present throughout the intervention, namely the intervention tools and the implementation of mental skills. The present study aims to investigate and evaluate the usefulness of intervention tools, and solely results pertaining to this point will be presented in the current article.

### Semi-structured interviews and data analysis

Semi-structured interviews were the main source of data for the present study (Kvale, 1997). Two different semi-structured interviews were conducted at the pre-testing and post-testing time-points. The semi-structured interviews had multiple aims. The first aim was to evaluate the intervention tools, while the second aim concerned assessment of participants' need for psychological skills, and the usefulness of these skills. Subsequently, in order to investigate the usefulness of the intervention tools, questions concerning the use of communication, performance profiles, group/individual meetings, iPads and electronic practice journals were assessed on four occasions throughout the intervention process in addition to the interviews.

Thematic analysis was applied analyzing the data (Guest et al., 2012). Thematic analysis identifies, analyze, and report themes in the data (Braun and Clarcke, 2006; Guest et al., 2012). Data analysis was conducted using NVivo for mac version 10.2.1.

### Intervention tools

#### Questionnaire

In order to assess the participants' strengths and weaknesses toward their awareness in relationship to instrument practice, performance preparation, and mental aspects of music creation, three previously validated questionnaires were translated into Norwegian and adapted to the context of the instrument practice. The original scales had been constructed for performance contexts such as education and sports. The questionnaire operated as a foundation for performance profiling throughout the intervention to help participants gain a multidimensional view of their current situation. The adapted questionnaire was based on the Self-Regulation Scale (Toering et al., 2012), Chronbachs' alpha between 0.73 and 0.85, Achievement Goal Questionnaire (Elliot and McGregor, 2001), Chronbachs' alpha between 0. 83 and 0.92, and Athletic Coping Skills Inventory (Smith et al., 1995), Chronbachs' alpha of 0.87. The adapted questionnaire consisted of 86 items intended to measure students' self-regulatory skills, psychological skills, and goal orientations. The questionnaire had eight subscales assessing planning and goal setting, motivation, self-efficacy, achievement motivation, use of mental skills, time management, self-reflection, and background information. All the subscales were scored using a five-point Likert scale. Each item was scored from “1 = Never” to “5 = Always” or “1 = Strongly disagree” to “5 = Strongly agree with the exception of background variables.

#### Performance profiling

**Figure 1:**
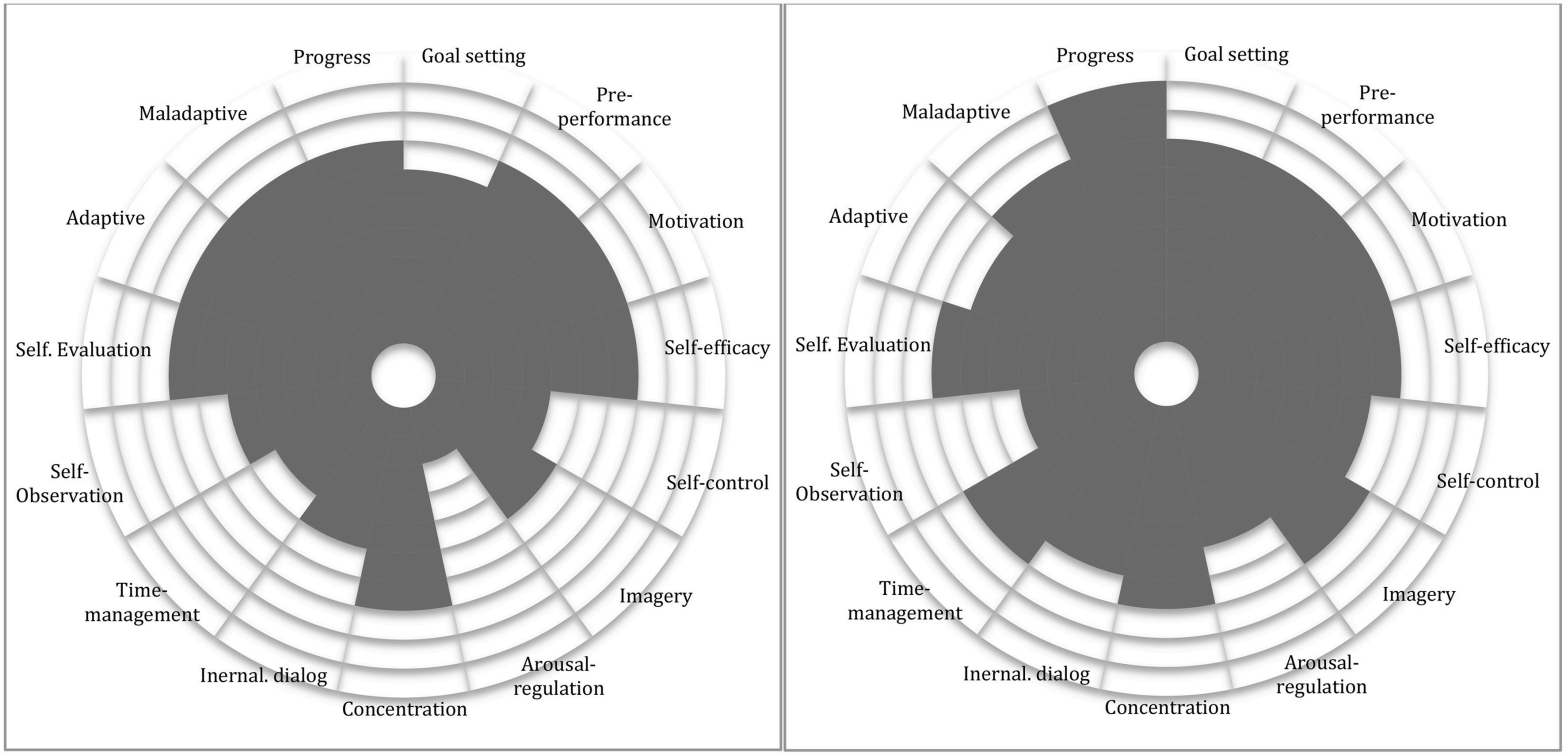
**Marcus' performance profiles (left, before; right, after)**. The shaded areas in the performance profiles below indicate the participants' desired outcome scored from 1 to 10. E.g. Marcus has scored 6 out of 10 points in the first assessment on goal setting on the left figure below.

**Figure 2:**
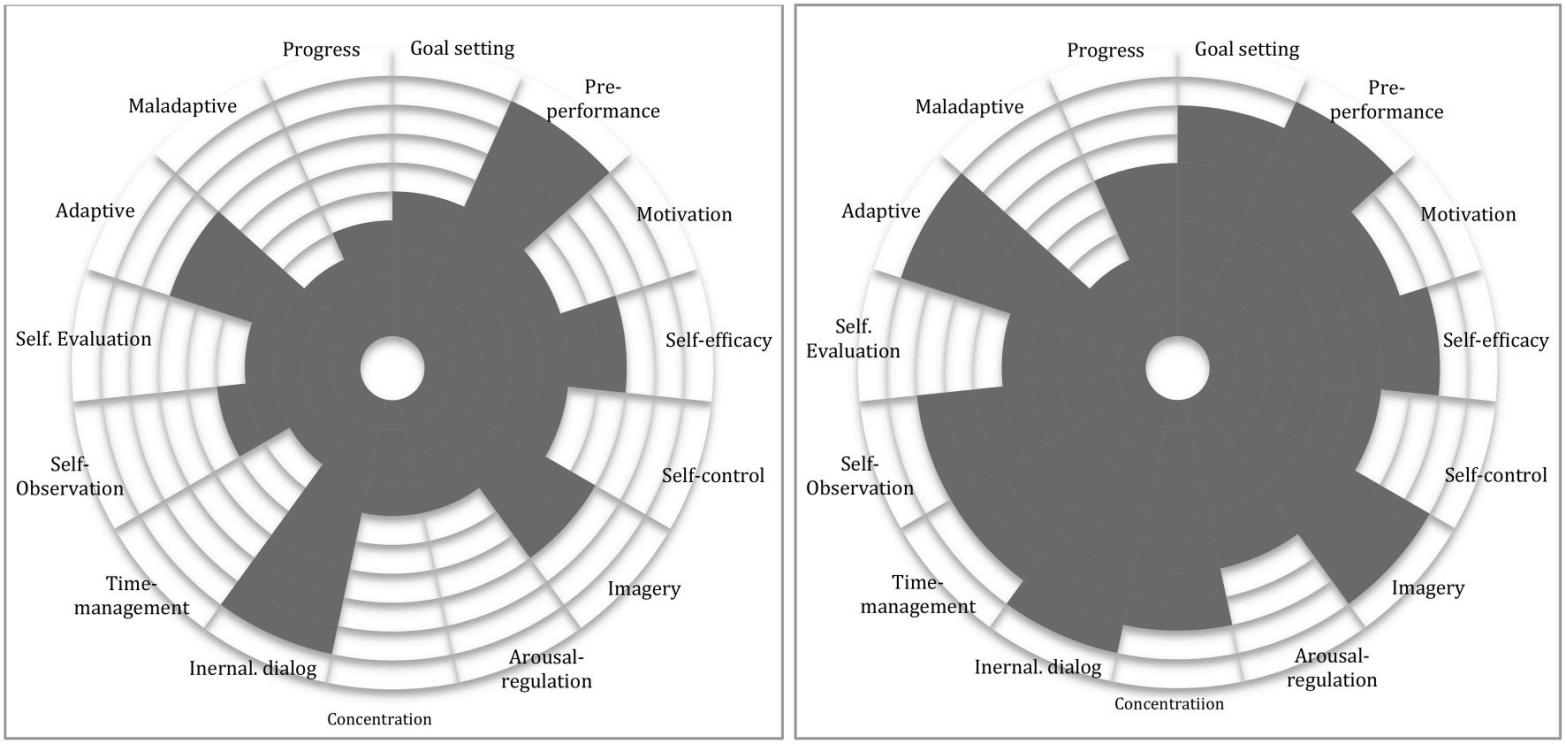
**Rita's performance profiles (left, before; right, after)**.

When constructing a PST program, multiple components are to be considered such as length of time, frequency of meetings, communication, work constellations, and focus areas among others. However, perhaps the most important aspect of planning PST interventions is to always keep in mind the uniqueness of each individual attending the program (Andersen, 2000; Hays, 2009). In the present study, the uniqueness of each individual was considered through performance profiling (Figures 1, 2). The profile consisted of categories derived from the questionnaires' sub-scales; goal setting, pre-performance, motivation, self-efficacy, self-control, imagery, arousal-regulation, concentration, internal dialog, time-management, self-observation, self-evaluation, and adaptive/maladaptive coping. The performance profile gave both the student and the tutor valuable information for further collaborative evaluation on where to start working (Mellalieu and Hanton, 2009; Weinberg and Gould, 2011). In this study, the performance profile was used to identify important individual PST goals. The profile was computed before and after the intervention providing information on participants' individual progress.

#### iPads and electronic practice applications

**Figure 3:**
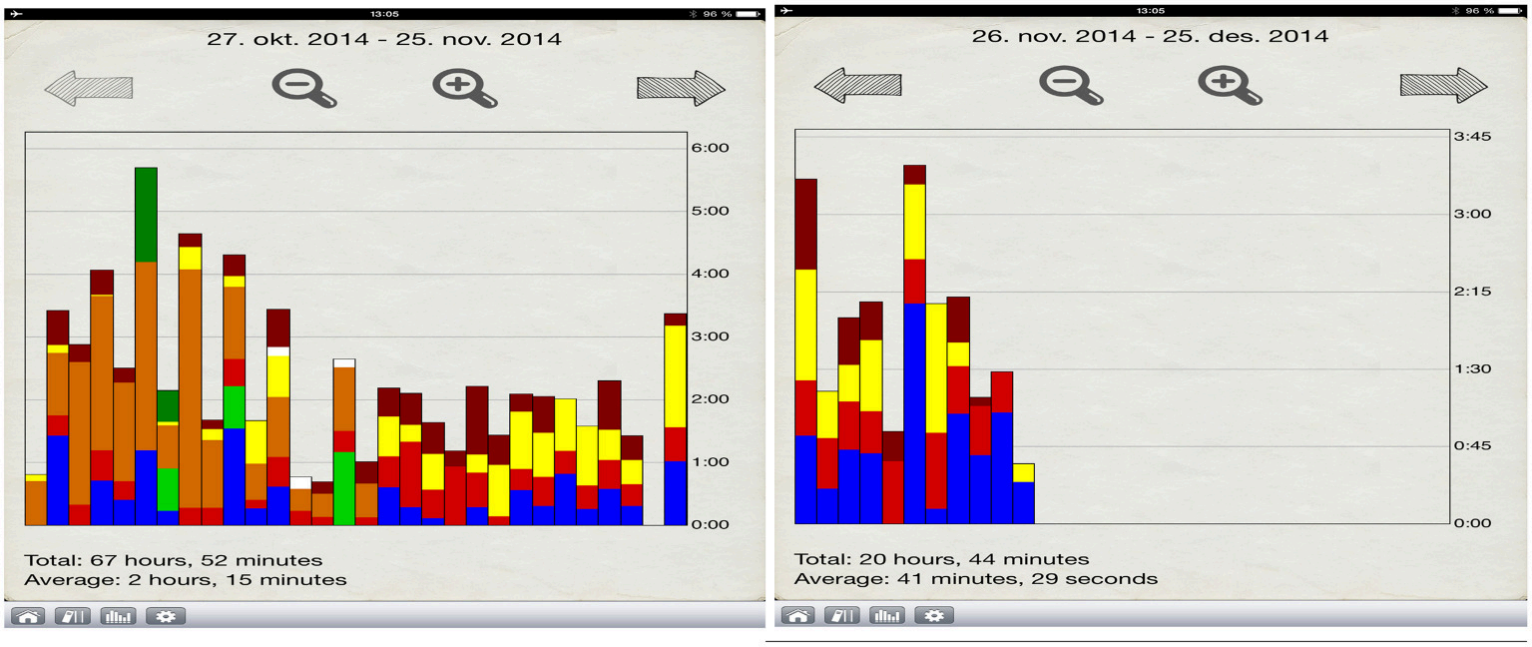
**Examples of the applications set up for time spent on multiple pieces/tasks throughout the intervention**.

The present study used the iTunes-based application Music Journal during the intervention. The application was installed on iPad Minis, which were handed out to the participants at the first meeting. The electronic practice journal recorded the total instrument practice time, along with the students' goals, practice time spent on various pieces, and goal achievement (Figure 3). In addition, the students wrote notes about their general experiences of the intervention program.

## Results

The present case study aimed to gain greater insight on how to develop PST-interventions for music students. The program evaluated multiple intervention tools including the use of questionnaires, performance profiling, iPads, electronic practice journals, the usefulness of individual and group work and different ways of communication, such as non-controlling use of language enabling autonomy and self-reference.

### Questionnaire and performance profiling

The use of the questionnaire in combination with follow-up semi-structured interviews provided the participants and the researchers with rich information about the students' instrument practice routines and level of performance. Interviews clarified contrasting answers to similar questions, and yielded specific and in-depth knowledge about key practice issues.

The first participant, Marcus, a violinist in his fourth year honor program at the academy, expressed his eagerness to begin the PST program. The first questionnaire suggested that he lacked structure and took an intuitive approach to instrument practice. Further in-depth interviews provided additional information about his experiences of performance profiling. Semi-structured interview confirmed most of the answers recorded by the questionnaire.

The performance profile provided the researcher and the participants with useful visual information concerning strengths and weaknesses in their instrument practice, as well as feed-forward regarding ongoing work. After having seen the performance profile during the first interview, Marcus was enthusiastic about the specific information offered by the profile, increasing his self-awareness and motivation to set specific goals.

*Marcus: Wow, I am really inspired about starting to work on what I just have discovered as my main weaknesses. Yes, I find this profile truly helpful for understanding what I need to focus on. Nobody has given me this kind of information before*.

The second case, Rita, also lacked structure and took a similar intuitive approach to her piano practice. In addition, the performance profile suggested that she was unspecific, impatient, and unconscious of time management during practice sessions. The first semi- structured interview confirmed what Rita had revealed in the questionnaire.

*Rita: Basically, I tend to plan my practice while being in the practice room with the instrument in hand. I basically play through and give attention to problem spots that appear. I always start with technique exercises, so I tend to go through a formal plan while warming up. I do not reflect upon the completed work after finishing my practice*.

During the first week Rita received her performance profile, she was interested, yet not surprised about, her weaknesses and strengths.

*Rita: I am actually not surprised about the results of this profile, but it even makes me more aware that this has to be taken seriously, which I have not managed in the past. I look forward to starting to work deliberately with goal setting and some planning*.

Both cases left the first session with a noticeable sense of optimism and ease after having set self-referenced goals for themselves. Both participants expressed motivation and optimism toward the continuation of the program. During the following individual session the week after, Marcus had already organized his practice differently.

*Marcus: I find it remarkable that one might use such a small amount of time on the various parts practicing the chunks slowly and thoroughly*.

In the Schumann, which was his main focus piece, Marcus had scanned through the work finding various focus points he had set working goals for and that he had started to give attention.

*Marcus: It is wonderful to know that it is only those parts I need to practice in the movement, and that I did not need to waste time starting from the beginning and randomly correcting some occurring mistakes*.

Both cases found this way of practicing much more time-efficient. The participants' performance profile had revealed that they lacked self-control, planning skills, and time management. These were the first aspects that both wanted to address. The performance profile helped both participants with planning and goal-setting, as well as increasing their motivation with the help of self-assessment strategies offering them a sense of ownership over the whole working process (Deci and Ryan, 2000).

Performance profiles are listed and illustrated in the Supplementary Material.

### iPads and electronic practice applications

During the first meeting, the participants were given a demonstration and instructions on how to use iPads and the Music Journal application. Participants expressed enthusiasm about the application, and immediately started to write down short and long-term goals.

Throughout the intervention, the application turned out to be a valuable tool for planning, keeping track of individual practice and repertoire, and time management. Participants explored how to use the video camera in order to record their practice sessions. Rita used the application rather consistently and said that she was amazed to discover how little time she spent on her individual practice. Subsequently, she noticed how much time she spent on pieces other than those from her solo repertoire. These points were clearly expressed in the post-interventions interviews.

*Rita: I have the same experience, I have never managed to maintain consistency, and I believe that the application was very helpful in showing how much I actually practice*.

*Marcus: To me it has been very helpful, especially using the application, I felt that I had something concrete to follow, knowing exactly what I was going to do on a daily basis was really inspiring and helpful*.

Furthermore, the music practice application also had a diary function that the participants used throughout the intervention. They reported that using the diary had made them reflect and evaluate themselves in a new way that made it much easier to attribute sources of success and failure. They also reported that they had found it challenging to use the diary on a daily basis, but when applied they felt that it enabled them to gain a greater insight on their overall development. However, both students struggled to maintain consistency in using the diaries. Both students reported that it took a great deal of volition after having finished a full day of practice to sit down to reflect and evaluate their day. However, they expressed that the extra time spent on after-practice reflection and evaluation was useful and necessary.

From a research perspective, the application allowed to gain precise information on how, what, and why the students practiced. In addition, the application stored all information in both quantitative and qualitative forms with the help of the journal and the statistical registration of practice. The iPads were convenient for recording and filming interviews as well.

### Group and individual meetings

Both participants were enthusiastic about the combination of group and individual meetings. Participants reported that they found these meetings necessary to allow them to focus individually and collectively on specific issues and solutions concerning the organization and execution of instrument practice.

*Rita: I very much enjoyed it; I feel that it is great to have something besides the interpretation classes with all the other students. In this small group I can actually discuss and play in a slightly more friendly, less frightening atmosphere than playing directly for all the professors and the other students, so yes, it was a good exercise in performing and applying the mental techniques*.

*Marcus: I believe that it was great having this small group, and not so frightening. I thought that in a group I might be a little more nervous about where to start. But it was great to experience that other students also have their problems and issues with practice. This makes it more human and transparent than a formal interpretation class setting. In the more formal setting one might believe that all the others are perfect, without any problems similar to your own. Through these sessions, I clearly learnt that this is not the case. Working in this type of group, where one openly discusses one's practice habits and strategies, has certainly given me a new dimension to my understanding of practice and performance*.

Both participants expressed a need for weekly individual meetings.

*Marcus: I believe that it has worked well up to now, but it would have been useful to have an individual lesson shortly after the group session, because one does not always feel like talking about all sorts of personal issues in front of other people*.

Rita: For me it was great to work individually, since we managed to focus more specifically on personal goals and discuss precisely how to work. In the group sessions, others were present and this affected me being open about my practice issues. The individual meetings were of great help since somebody helped me understand explicitly why, how, and what to practice. Since I have had issues concerning being self-disciplined for years, having the opportunity to reflect on specific techniques has made me both more aware and more motivated toward my practice. I need to become acclimatized with this way of working…

*I wish I could have had more than two individual meetings per month. I believe that the best idea would be to have an individual follow-up meeting after the group sessions. I missed having the opportunity to discuss and plan my practice under your “[the researcher's]” guidance in the weeks we had group lessons*.

Viewing the students' progress over time, having 2 weeks between the individual sessions seemed to have affected the students' continuity. Especially, at the beginning of the program, it turned out that two individual meetings per month were insufficient. The individual work was found to be rather time consuming, but useful in relation to the participants' acquisition of mental techniques. In sum, the individual sessions laid a foundation for the participants' directedness and volition, and consequently made them more proactive in their practice approach.

Finally, the group sessions also turned out to be a highly useful setting for exercising and mocking various mental skills in addition to interpretation classes and other performance settings. Moreover, the participants were able to use this small group setting as an opportunity to gain performance experience within a medium pressured performance context making them more robust and prepared for more demanding performance settings.

### Forms of communication

In relation to SDT' claim that non-controlling language enables psychological need satisfaction and autonomy, post-interview revealed the following:

Rita: I need to be told explicitly how to plan my sessions. When I managed the entrance audition, I was inspired and satisfied with my performance, but I have never been told this before. During my lessons with the professor, we mostly discuss the music, technique, fingering, and phrasing, but never how to plan my practice sessions and set goals. Before, I tended to practice without my head turned on. I really liked receiving concrete messages and instructions that revealed I should actually engage in a more efficient method of practice. So, I needed somebody to tell me things like: go ahead and just do it, this week you will really go ahead and accomplish your tasks, be determined and enjoy it!

*Marcus: Well, I do believe that both approaches are good. I believe that answering the open-ended questions has made me more independent and aware, including after completing the intervention. But at the same time I find it very inspiring to receive clear directions and advice*.

The students' motivation for playing and becoming musicians influenced their receptiveness toward the intervention. Constantly alternating between inductive and deductive means of communication seemed to influence students' implementation of the program positively. Students were abled to set their own goals by asking open-ended questions, giving them a sense of ownership and autonomy toward their practice and development (Deci and Ryan, 2000). When using direct communication forms, the main investigator always provided the student with a clear rationale for why a certain action was important. On a few occasions, the main investigator challenged the student on finding out the best course of action. Participants were clearly most active during the intervention, enabling personal identification with activities of focus.

When asked about the overall experience of the program, Marcus responded:

*I enjoyed learning how to be more structured in my instrument practice, not approaching my instrument practice by doing what I use to call “panic practice,” and not practicing last minute before my lessons or interpretation classes*.

During the whole intervention, Rita had a heavy workload in addition to her studies, which resulted in her generally playing one or two concerts per week.

Rita: I have discovered that I have made myself too busy in recent years by accepting a lot of jobs outside the conservatory. I would like to try to give myself a little more space and spend more time on my solo repertoire. I am not sure if I would have come to this conclusion without participating in this project. I am very inspired to continue and get more time to dedicate myself, in order to implement what I have learnt during the intervention…

*I would like to continue working on, planning and goal setting. I need to try to find a certain balance between the various activities I engage in. I realize that I need to prioritize my individual practice more than before*.

Marcus seemed to have benefitted from learning how to set multiple types of goals and expressed enthusiasm about continuing to set both general and specific goals for himself.

*Marcus: I would love to continue using goal setting the way I have learnt, I felt that it was very useful in preparing my practice sessions. I felt that I had much better control over what I was supposed to practice. Especially when I had a lot to do, I felt that it was a great tool to make my practice more efficient by focusing on things that I needed to practice, instead of just playing through*.

Even though the main interest in this study was the effectiveness of the intervention tools, the psychological skills training program seemed to initiate positive progress in the students' practice routines. Finally, the intervention seemed to have stimulated the students' interest in continuing to explore and implement psychological skills. Both Rita and Marcus exhibited strong motivation toward the end of the intervention. Marcus was especially pleased to be selected as a substitute for the local philharmonic orchestra after having implemented psychological skills including audition simulation, imagery, and arousal regulation as part of the audition preparation.

The study also highlighted the importance of students' availability in terms of time. For instance, Rita turned out to be highly active as a concert performer, and engaged in a wide variety of jobs in addition to her music studies. As a result, she expressed frustration about not having enough time to fully focus her attention on her music studies and the intervention. Thus, students' availability is also an important consideration in order to gain the best possible results in future interventions. After finishing the intervention, Rita decided to reconsider her overall workload in relation to her values and music studies.

## Discussion

Intervention programs focusing on the enhancement of mental skills needs to be individually tailored (Andersen, 2000, 2005; Hays, 2009; Weinberg and Gould, 2011). A unique finding from the current study was the importance of the participants' understanding of their own capabilities, which could potentially influence their commitment to the program. After completing the performance profile, both participants expressed a desire to promptly start setting goals for their practice and investing resources to reach them. This correspond to the process of gaining a rational understanding of why one ought to engage in an activity (Deci and Ryan, 2000). Within SDT, this type of motivation is termed identified regulation. The participants set goals that resonate with their personal beliefs. The investigator's conscious support of the participants' need for autonomy has likely and positively influenced the students' motivation throughout the intervention. A less personalized approach would have likely produced poorer forms of motivation in participants requiring greater external forms of motivation to achieve similar levels of commitment but leading lower levels of integration of practice behaviors over time (Deci and Ryan, 1985, 2000). These findings need to be replicated with larger populations. However, it is clear that adapting a motivational approach based on SDT provides useful framework to insure an adaptive climate to implement PST for music students.

Previous studies have suggested that the solitude experienced by performance music students can be detrimental (Jørgensen, 1996; Atkins, 2009). SDT claims that the fulfillment of the basic psychological need for relatedness is important to nurture intrinsic forms of motivation. Socialization through group sessions in which the participants could identify with and relate to one another was important for meeting their psychological need for relatedness. Marcus revealed that, some years ago, he had collaborated with other violinists by meeting at school early in the morning, taking agreed breaks, and pushing each other to practice. Marcus experienced this as highly effective and pleasurable because he could identify with his peers and at the same time manage his time schedule and maintain his personal need for instrument practice. The current intervention produced similar feelings. Consequently, combining the use of group and individual work is important when designing PST-interventions for music students.

Working on deliberate practice techniques such as planning and goal setting had an impact on Rita and Marcus' focus toward task-relevant aspects in their practice. Goal setting seemed to influence the participants' level of concentration. This indicates that setting specific working goals promotes commitment and involvement in the task at hand (Locke and Latham, 2002). Furthermore, this work on goal setting was greatly facilitated by the use of the iTunes-based application Music Journal. Findings revealed that the application not only worked as a practice journal for goal setting and reflection, but also provided both the researcher and the participants with precise information concerning the amount of accumulated practice, as well as the amount of time spent on diverse pieces of music and techniques. The iPad turned out to be a practical tool for self-assessment and keeping track of progress, as it allowed the students to film their performance, with sufficient sound quality.

During the intervention it became evident that the students had some prior knowledge of planning instrument practice. However, this appeared to be limited to theoretical knowledge, which students had not actively integrated into planning their individual work on their instruments. The students seemed pleased to finally be in a context in which the main goal was to prepare their practice. Earlier studies on practice have revealed that professors tend to believe that their students have an independent understanding of how to organize their practice (Jørgensen, 2000; Burwell and Shipton, 2013). However, current study findings clearly confirm music students' need for support toward planning, goal setting, and maintenance of their instrument practice routines. In this process, both participants found clear instructions and expectations helpful. In addition, communication through which the participants tailored their own solutions and plans generated higher levels of motivation and commitment. Participants' general motivation for being involved in the various elements of the intervention seemed to correspond with what SDT terms *integrated regulation*. The distinction between controlled and autonomous regulation is closely associated to what extent an external form of motivation is integrated into the person's sense of self. Moreover, the style of communication, deductive or inductive, is believed to be useful as long as the participant intrinsically identifies with the subject being communicated. These findings ought to be replicated and tested in future research with music students.

The research method is subject to important limitations. First, the evaluation of the intervention tools might have been made more consistent by using an inventory approach assessing participants' experiences of the tools evaluated. This might have given more systematic quantitative measures of how the participants perceived the intervention tools evaluated. However, it was disregarded, a questionnaire approach with only two participants probably would have produced similar results to semi-structured interviews, but less specific. Secondly, the study was primarily designed to generate information about the construction of a larger inter-disciplinary study on psychological skills training for musicians. It highlighted the difficult task of combining evaluative and interventional aspects simultaneously. Hence, the usefulness of the implementation of PST techniques may have been negatively affected by the focus on assessing the intervention tools and vice versa. Consequently, future research might exclude, or downplay the role of implementation of PST in order to fully assess intervention tools effectiveness. Interventions solely focusing on PST implementation are most likely to yield more complete descriptions of the effects of PST interventions.

## Conclusion

The goal of the present research was to gain knowledge about components that are beneficial to interventional research within the instrument practice field of music. The case study found evidence to support the importance of participants' personal interest and engagement for the intervention. Clearly, personal interest is a variable that needs to be considered when selecting participants for future psychological skills interventions in the music context. This study provided students with performance profile information based on questionnaire self-assessment, a substantial source of motivation and commitment toward continuing work (Weinberg and Gould, 2011). Consequently, a second central implication is that participants who used self-referenced goals, which included a clear rationale for engaging in self-enhancing techniques, are more likely to show interest in initiating such work. Combining group and single PST-sessions turned out to strengthen the overall delivery of the program enabling participants' needs. It also leads to open communication and performance training with the other participant. Using iPads and electronic practice journals enabled valuable feedback and feed-forward for both the participants and the implementer. The journal provided participants with a valuable tool to set specific goals and evaluate themselves on a daily basis. This way of working positively affected participants' motivation and perception of instrumental progress.

Finally, the participants reported that they best received the psychological skills intervention when the researcher altered between a deductive determined way of communication comprising rationales for involvement and development, and an inductive open-ended communication approach generating free associations. Moreover, procedures deriving from SDT were a noteworthy source of motivation and enthusiasm due to the theory's explicit emphasis on inherent psychological needs. However, future theoretically based research is needed in order to provide more substantial indications of SDT-based framework's potential to explain motivation in the teaching, learning, and practice of music.

### Conflict of interest statement

The authors declare that the research was conducted in the absence of any commercial or financial relationships that could be construed as a potential conflict of interest. The reviewer Terry Clark and handling Editor declared their shared affiliation, and the handling Editor states that the process nevertheless met the standards of a fair and objective review.
