# Supplementary material for: Foundations of Intervention Research in Instrumental Practice
Source: Front Psychol. 2016 Jan 22;6:2014. doi: 10.3389/fpsyg.2015.02014 (PMC4722117; doi:10.3389/fpsyg.2015.02014)
Supplement: Supplementary file 1 [file DataSheet1.docx]

**Questionnaire**

**Welcome to a survey on instrumental and mental practice in music.**

The questionnaires aim is to gain knowledge about music students practice habits and self-regulation skills. The questionnaire contains in all 87 questions and will take 5 to 10 minutes to complete. I would truly appreciate if you could answer the questions as truthfully as possible. All answers and information will be treated confidentially. The technical implementation of the survey is conducted by Survey-exact. The researcher will be given data from Survey-exact, which is linked to email or IP addresses. All this information will be made anonymous when the project is reported and completed, by 01.09.2016

Many thanks for your interest and help. Best wishes!

All the questions in this survey concern multiple elements of instrumental practice and performance. I would appreciate if you could answer the questions as truthfully as possible and in relation to your currant practice and performance situation.

| I always set concrete long-term goals for myself. |
| --- |
|  |
| In relation to my long-term goals, I set specific short-term goals for my practice. |
|  |
| I usually do not set specific goals for my practice in advance, the goals comes to mind with the instrument in hand. |
|  |
| I utilize a practice journal/application during planning and evaluation of my instrumental practice. |
|  |
| On a daily or weekly basis, I set very specific goals for myself that guide what I do. |
|  |
| I set very specific goals and know what it takes to reach them. |
|  |
| I imagine the parts of a problem I still need to complete. |
|  |
| While doing a task, I ask myself how well I am doing. |
|  |
| I clearly plan my course of action to solve a problem in my instrumental practice. |
|  |
| I develop a specific plan for the solution of a problem in my practice. |
|  |
| The planning of my instrumental practice happens intuitively with the instrument in hand. |
|  |
| I have my own performance/concert plan worked out in my head long before the performance begins. |
|  |
| I feel confident that I will perform well. |
|  |
| I consciously prepare for concerts/auditions through mocking/simulating the concert/performances for colleagues,  friends and others. |
|  |
| I strongly believe that I have what it takes to accomplish what I start working on. |
|  |
| I can solve most problems if i invest the necessary effort. |
|  |
| I always manage to solve difficult problems if I try hard enough. |
|  |
| When I am confronted with a problem during practice, I usually find several solutions. |
| I believe that I am able to become more consistent and goal-directed in my instrumental practice. |
|  |
| If I persist on a task, I´ll eventually succeed. |
|  |
| I am unmotivated because I do not achieve the progress that I am hoping for. |
|  |
| I am unmotivated because I feel that I am not consistent and directed enough during instrumental practice. |
|  |
| I am motivated towards reaching the goals that I set for myself. |
|  |
| It means a lot to me to reach the goals that I set for myself. |
|  |
| I usually practice very deliberately towards specific tasks. |
|  |
| I desire to master the material I am working on regardless of other student’s work and progress. |
| While practicing, I am only preoccupied with the task at hand. |
|  |
| It is important to me to perform equally well or better than my peers. |
| I am motivated by the thought of outperforming my peers. |
|  |
| I am afraid of performing below the other students´ standard. |
|  |
| I usually worry about difficult parts of a piece while performing. |
|  |
| I compare myself to other students and tend to avoid performing below their standard. |
|  |
| I often think to myself "what if I am not prepared enough for this performance". |
|  |
| While practicing, I tend to worry about not doing the right things. |
|  |
| I tend to loose focus towards task while practicing due to a desire to master the task immediately. |
| I usually practice in the original tempo with the same expression as during concert. |
|  |
| I am tempted to prematurely practice new pieces in the original tempo. |
|  |
| While approaching new works of music, I am taken away by the expressive character of the music,  and I practice with full expression. |
|  |
| I keep difficult passages within a slow tempo in order to master them. |
|  |
| I am unfortunately not consistent enough with my instrumental practice. |
|  |
| I often use imagery in relation to instrumental practice. |
|  |
| I often use imagery in relation to concerts and performances. |
|  |
| I always prepare my level of arousal before/while practicing. |
|  |
| I often get overly tense during concerts and badly influenced by this. |
|  |
| When I feel myself getting too tense, I can quickly relax my body and calm myself. |
| It is easy for me to direct my attention and focus towards what I am practicing. |
|  |
| It is easy for me to keep distracting thoughts from interfering with my instrumental practice. |
|  |
| I easily get distracted while practicing. |
|  |
| When I am performing, I can focus my attention and block out distractions. |
|  |
| I consciously engage in positive thinking while practicing. |
|  |
| I usually communicate negatively with myself while practicing. |
|  |
| I think about and imagine what will happen if I fail or screw up before concerts. |
|  |
| I maintain emotional control regardless of how things are going for me. |
|  |
| I plan how long I should practice before taking brakes. |
|  |
| I follow a well-established plan for how long I should practice. |
|  |
| I am currently not managing my time of practice. |
| I do often practice for longer than 60 minutes before taking brakes. |
|  |
| I have a specific plan for how long each practice session should last. |
|  |
| I take practice-brakes when I feel my body is starting to hurt. |
|  |
| I have experienced pain related to performance and instrumental practice. |
| 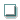I have experienced pain  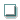I have experienced some pain  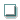I have not experienced pain |
| I do currently experience pain related to instrumental practice and performance. |
| 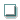I experience pain  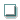I experience some pain  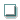I do not experience pain |
| I check my accuracy while progressing through a practice task. |
| I observe my practice from an analytical perspective while practicing. |
|  |
| I check how well I am doing when I solve instrumental practice tasks. |
|  |
| I keep track of my progress over time. |
|  |
| When having practiced something during longer periods, I look back to see if I did the right procedures. |
|  |
| I am generally good at evaluating my instrumental practice and finding adequate solutions. |
|  |
| When things turns out badly during concerts, I try to think about how I can do things better next time. |
|  |
| I think through past performance experiences to understand new practice ideas. |
|  |
| When I´m not achieving the desired results, I carefully search for plausible reasons that leads to new adequate goals. |
|  |
| I perform well because I am well prepared. |
|  |
| When things turns out badly, I often get fed up and loose faith in myself. |
|  |
| When the performance turns out badly, I try to avoid screwing up at the next performance. |
|  |
| I might give up a task if it does not turn out as expected after having invested a lot of work in it. |
| I believe that my current progress reflects to the amount of hours spent on practicing. |
|  |
| I believe that my progress is poor due to me being too impatient. |
|  |
| I believe that I have generally little progress according to the amount hours spent on instrumental practice. |
|  |
| Are you in the Master or Bachelor program? |
| 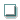Bachelor program  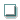Master program  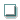Other program |
| Do you attend the music performance, music education, music therapy, composition program, or other program? |
| 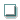Music performance program  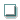Music education program  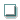Music therapy  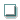Composition  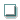Other program |
| Do you attend the program of jazz, folk music, or classic music? |
| 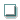Jazz  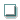Folk music  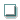Classical music  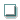Other |
| Which category of instruments do you belong to? |
| 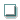Stringed instruments  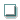Key-instruments  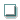Brass  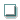Woodwinds  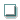Percussion  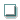Singing  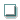Guitar/stringed instruments |
| Approximately how many hours do you currently practice a day? |
| 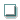Less than one hour  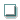1 to 2 hours a day  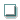3 to hours a day  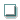2 to 3 hours a day  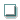4 to 5 hours a day  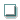5 to 6 hours a day  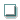6 hours or more a day |
| I started playing my instrument aged. |
| 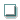3-4 years old  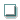5-6 years old  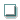7-8 years old  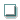9-10 years old  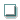11-12 years old  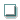13-14 years old  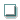15 years old or older |
| Select your gender. |
| 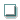Female  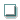Mail |
| Du you work beside your music studies? |
| 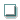I do not work  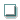I work beside |
| How much time do you have for instrumental practice? |
| 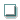Little time  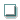Some time  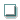Enough time  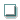Plenty of time |
| Do you use your time efficiently? |
| 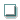Very Inefficiently  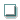Inefficiently  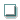Partly efficiently  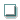Very efficiently |
| Which part of the world do you come from? |
| 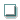Norway  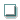Northern Europe  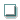Eastern Europe  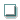Southern Europe  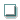Africa  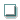Asia  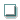The USA or Canada  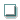Latin America |

**Semi-structured interview:** Interview guide, 27^th^ of October 2014

The aim of this interview is to gain knowledge about the participants’ needs and current practice behaviour prior to implementing psychological skills training. The Interviews were individually tailored in order to address questions from the completed questionnaire, which was found to be of particular significance to each interviewee.

General background questions:

1) First of all, what expectations do you have prior to starting the program?

- Is there something in particular that you are hoping to achieve during this program?

2) Do you have any personal whishes or requests that you would like to work on?

- How do you expect to achieve the whish?

Personal follow-up questions based on completed questionnaire:

Pilot 1:

1. The questionnaire shows that you seldom plan or manage your practice sessions before you start practicing, have you ever tried to do so for shorter periods before?
2. The questionnaire shows that you apply various strategies dealing with how to solve practice tasks, could you give me some examples of this?
3. How exactly do you plan your practice sessions?
4. You have received a high score on questions concerning planning of concerts and performances; would you say that your planning correspond with your performance achievements?
5. Questions 16, 17 and 18 from the questionnaire reveal that your actual performance achievements do not resonate with your expected performance achievements. Could you please try to imagine a situation in which this is the case and give an example/examples of this?
6. What is your foundation for achieving positive progress and success?
7. To what extent do you manage to pay attention towards one task at the time?
8. Could you tell me about how you organize your practice when starting on a new piece of music?
9. Do you feel motivated about the pieces that you are planning to play during the intervention?
10. Do you ever pay attention to physical arousal while practicing your instrument?
11. Could you please imagine yourself practicing, how do you solve difficult tasks? - How do you communicate with yourself while solving difficult tasks?
12. Could you tell me how you manage your time of practice?
13. Do you ever experience that it might be difficult to take brakes while practicing?
14. Do you have any kinds of physical pain, injuries due to instrumental practice?
15. How do you keep track of your progress?
16. What motivates you in your individual practice?
17. Do you have any specific goals for the future that motivates you?
18. Have you ever experienced that your short-term motivation might damage your long-term achievements and motivation, or vice versa?

Personal follow-up questions based on completed questionnaire:

Pilot 2:

1. The questionnaire shows that you seldom plan or manage your practice sessions before you start practicing, have you ever tried to do so for shorter periods before?
2. The questionnaire shows that you apply various strategies dealing with how to solve practice tasks, could you give me some examples of this?
3. Have you ever tried to use a practice journal or practice application previously?
4. What exactly determines what you find important in your instrumental practice?
5. How exactly do you plan your practice sessions?
6. You have received a high score on questions concerning planning of concerts and performances; would you say that your planning correspond with your performance achievements?
7. Could you explain what that demotivates you in the goals you or others set for your instrumental practice?

-What types of goals do you believe might increase your motivation?

1. Have you ever felt motivated before due to planning and maintaining good structure in your practice? - What did you do differently then compared to now?
2. What is your foundation for achieving positive progress and success?
3. What makes you enjoy your practicing, how does this happen?
4. Could you tell me about how you organize you practice when starting on a new piece of music
5. Could you tell me about how you organize you practice when starting on a new piece of music
6. Do you feel motivated about the pieces that you are planning to play during the intervention?
7. Do you ever pay attention to physical arousal while practicing your instrument?
8. Could you please imagine yourself practicing, how do you solve difficult tasks? - How do you communicate with yourself while solving difficult tasks?
9. Tell me about how you manage your time when practicing?
10. Do you experience that it might be difficult to take brakes while practicing?
11. Do you have any kinds of physical pain, injuries due to instrumental practice?
12. How do you keep track of your progress?
13. What motivates you in your individual practice?
14. Do you have any specific goals for the future that motivates you?
15. Have you ever experienced that your short-term motivation might damage your long-term achievements and motivation, or vice versa?

**Interview guide post semi-structured interview**

The aim of this semi-structured interview is to gain knowledge about the participants’ general experiences of being introduced to PST. Furthermore, the interview aims to gain knowledge about the various ways of working, both in-group and individually and if and how the students perceived the intervention tools, and if they are interested in continuing to using the techniques.

1. How did you experience the initial phase of the program in PST?

- Was there anything you liked/disliked in the beginning?

2. Could you please try to recollect memories about the whole course from the beginning to the end, could you please say something about how you experienced your own development during the past months?

- What was particularly good with the course (various elements)
- Thinking in retrospect, is there anything you would take away from the course?

3. Do you feel that the course has given you something to continue working on?

- Is there anything you would like to continue to use in your future practice?
- Is there anything that you would not continue working on after the course?

4. How did you experience working in group/performing for others?

- In what way?
- Have you learnt anything form discussing and reflecting music practice and performance with others within the group?

5. How did you experience the ambient in the group?

- Is this something to continue with in higher music education?

6. How did you experience the combination of group and individual work?

- Would you prefer more individual or more group work if you could begin all over again?

7. Have you gained more knowledge about yourself and music practice?

- What in particular have you learnt from this?

8. How are you approaching with the themes that you wanted to enhance?

- Do you have any strategies for developing them? How?

9. How did you experience to use the ipad as a practice tool?

- Have you continued to use the ipad throughout the whole program?
- How did you experience working on concrete goals on the ipad
- Is this something you would like to continue doing?
- Have you used the video-option on the ipad?

10. How has it been to work on goal setting?

- Which types of goals did you particularly enjoy to work on?
- Do you have any new goals for the future?
  - What goals? How will you apply them?

11. What is you experience of performance profiling?

- In what way was this helpful/unhelpful?

12. How did you experience the communication of the program?

- What type of communication do you prefer?

1. Instructional/demanding

2. Open-ended/free choice

3. A mix of both

Why?
